# Pep-Calc.com: a set of web utilities for the calculation of peptide and peptoid properties and automatic mass spectral peak assignment

**DOI:** 10.1007/s10822-016-9902-7

**Published:** 2016-02-24

**Authors:** Sam Lear, Steven L. Cobb

**Affiliations:** Department of Chemistry, Durham University, South Road, Durham, DH1 3LE UK

**Keywords:** Peptide, Peptoid, Calculated properties, Automatic mass assignment, ChemDraw export, Calculator

## Abstract

The ability to calculate molecular properties such as molecular weights, isoelectric points, and extinction coefficients is vital for scientists using and/or synthesizing peptides and peptoids for research. A suite of two web utilities: Peptide Calculator and Peptoid Calculator, available free at http://www.pep-calc.com, are presented. Both tools allow the calculation of peptide/peptoid chemical formulae and molecular weight, ChemDraw structure file export and automatic assignment of mass spectral peaks to deletion sequences and metal/protecting group adducts. Peptide Calculator also provides a calculated isoelectric point, molar extinction coefficient, graphical peptide charge summary and *β*-strand contiguity profile (for aggregation-prone sequences), indicating potential regions of synthesis difficulty. In addition to the unique automatic spectral assignment features offered across both utilities, Peptoid Calculator represents a first-of-a-kind resource for researchers in the field of peptoid science. With a constantly expanding database of over 120 amino acids, non-natural peptide building blocks and peptoid building blocks, it is anticipated that Pep-Calc.com will act as a valuable asset to those working on the synthesis and/or application of peptides and peptoids in the biophysical and life sciences fields.

## Introduction

Convenient and rapid access to calculated molecular properties is essential for researchers using and/or synthesizing peptides and peptidomimetics for biophysical or life sciences applications. Furthermore, the process of assigning peptide byproducts in mass spectra resulting from residue deletions or incomplete protecting group removal during a synthesis can be a laborious and time consuming process, and access to freely available automatic assignment tools is necessary to improve workflow and increase research efficiency. While a plethora of peptide and protein property calculation tools are accessible online, very few offer mass spectral peak assignment functionality, and for those that do this is often extremely limited.

While the ExPASy portal [1] acts as the most comprehensive protein property calculation resource for molecular biology, other more specific tools exist, such as ChemCalc [2], PredictProtein [3], IMSPeptider [4], POTAMOS [5], Top Pred [6], CheckMyMetal [7], AFAL [8] and a host of other peptide property calculation utilities [9–16]. Few of these are designed specifically with the synthetic peptide chemist in mind however, and furthermore, to the best of our knowledge, no freely available web services exist for the calculation of *peptoid* molecular properties or assignment of peptoid synthesis mass spectra.

We present a pair of web tools: Peptide Calculator and Peptoid Calculator, for chemical formula and molecular weight calculation of peptides and peptoids. In addition, both sites offer automatic assignment of mass spectral peaks to deletion sequences, metal ion adducts and protected byproducts, as well as the option to download structures in ChemDraw format for the sequences entered. Peptide Calculator can also give calculated values for isoelectric point and molar extinction coefficient (at 280 nm), as well as a plot of calculated *β*-strand propensity for the sequence. Both utilities are available at http://www.pep-calc.com.

## Features summary

### Sequence input

**Fig. 1:**
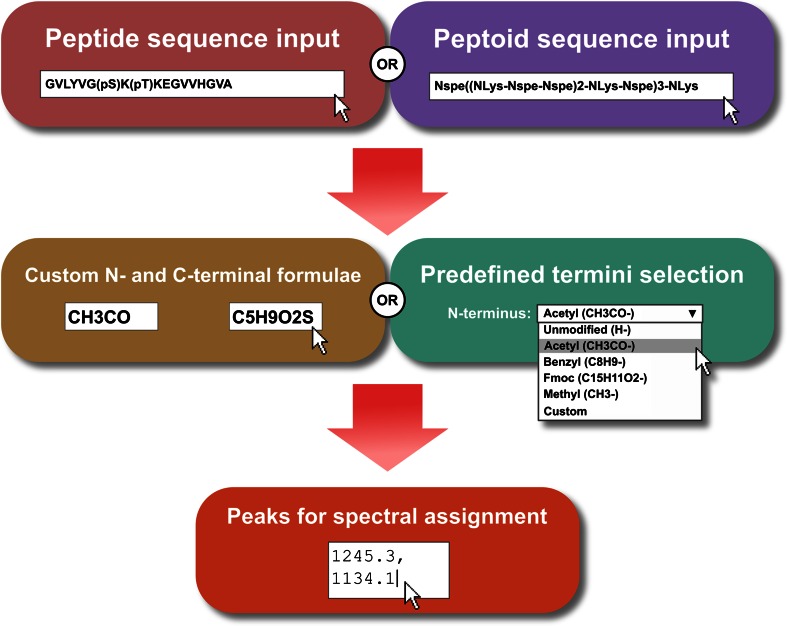
Summary of input options available for Peptide Calculator and Peptoid Calculator. Sequences can be specified using a large variety of residue types, and Peptoid Calculator also accepts input strings containing repeating sequence motifs indicated by *nested parentheses*. Termini formulae can be selected from available options and are also fully customizable. Optionally, *m*/*z* values can be specified for automatic peak assignment

Peptide and peptoid sequences up to 150 residues in length can be entered, containing any combination of amino acids or peptoid building blocks present in the database. For peptides, the input string may include any of the standard single-letter amino acid codes in addition to a number of ‘nonstandard’ residues (such as phosphoserine, *pS*), which must appear in parentheses within the string. An equivalent set of single-letter codes does not exist for peptoid building blocks, therefore Peptoid Calculator instead accepts a string of residue codes separated by dashes, without the requirement for multiple-letter codes to be enclosed in brackets. As peptoids can often consist of repeating motifs, Peptoid Calculator additionally allows parentheses to be used to indicate repeat sequences within the input string. Peptide and peptoid sequence input options are summarized in Fig. 1.

Both utilities also offer the option of specifying formulae for the N- and C-termini of the input sequence. These can be entered as a custom molecular formula string, or selected from lists of predefined formulae (Fig. 1). A full list of available residue types (showing residue code, molecular formula and molecular structure) and predefined termini available on Peptide Calculator and Peptoid Calculator is given on each site’s Help page.

A final (optional) input field can be used to specify *m*/*z* values belonging to singly-charged species in mass spectra, for automatic assignment to peptide or peptoid deletion sequences and/or adducts (described below).

### Calculated parameters

**Fig. 2:**
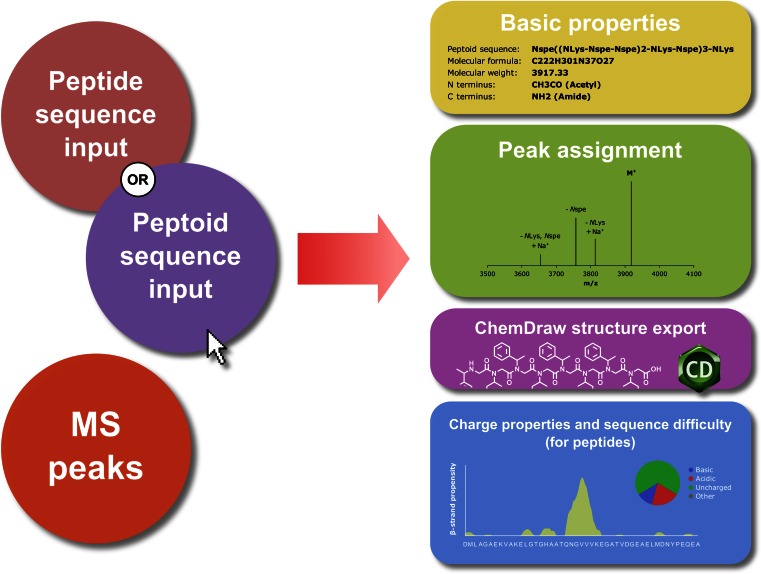
Both Peptide Calculator and Peptoid Calculator will output a number of basic calculated properties, in addition to a peak assignment and ChemDraw structure file for the sequence. A number of additional parameters are also provided for peptides, including estimated isoelectric point and molar extinction coefficient, as well as a graphical residue charge summary and *β*-strand contiguity profile

Both utilities will provide a molecular formula and calculated molecular weight for peptide/peptoid sequences entered, in addition to an automatically generated ChemDraw structure in .cdxml format (Fig. 2). A spectral assignment for the peptide/peptoid will also be given if *m*/*z* values were provided as part of the input.

**Fig. 3:**
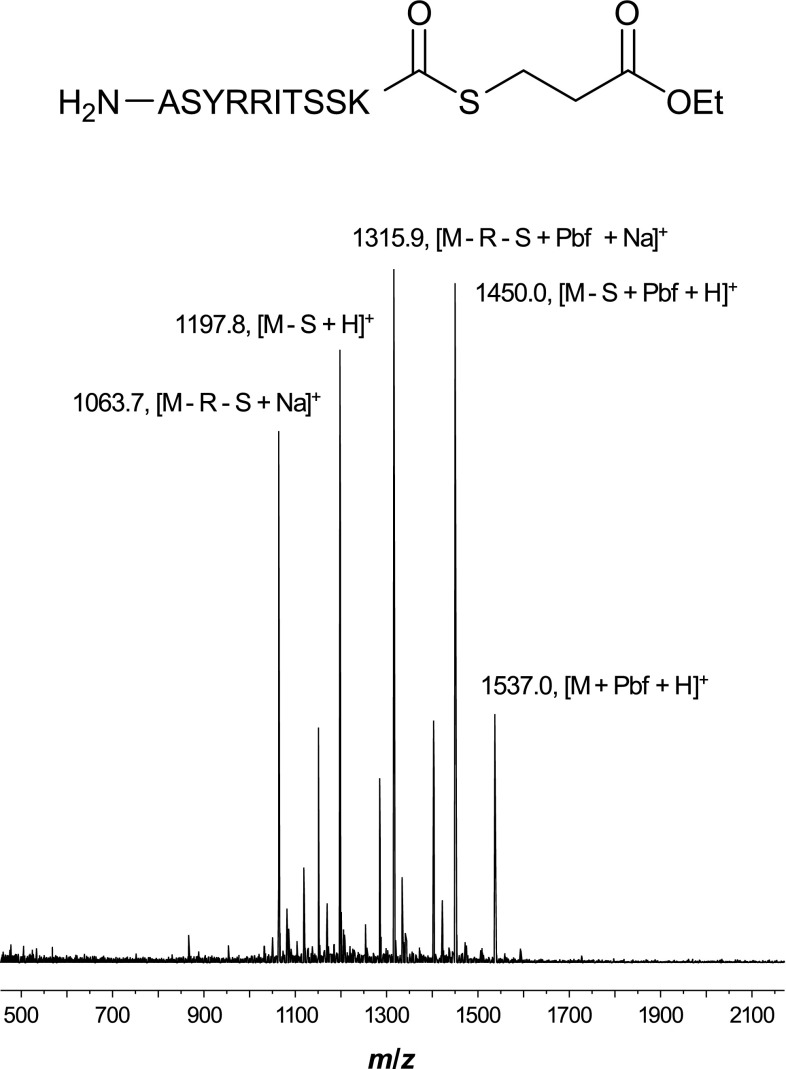
Example spectrum automatically assigned by Peptide Calculator (assignment is also available for Peptoid Calculator). A number of single- and multiple-residue deletions have been identified, in combination with sodiation and/or unremoved 2,2,4,6,7-pentamethyldihydrobenzofuran-5-sulfonyl (Pbf) protecting groups. The ethyl 3-mercaptopropionate thioester is available as a predefined C-terminus and can be selected during sequence input

An example of an automatic peak assignment is illustrated in Fig. 3 (assignment output shown in Table 1). A number of peaks are present in the spectrum and have been assigned to either deletion sequences (where one or more residues are missing from the target sequence), sequences with unremoved protecting groups, metal adducts or a combination of two or more of the conditions described. Peptide Calculator and Peptoid Calculator will attempt to assign any *m*/*z* values provided to either the target sequence or a formula containing single or multiple residue deletions, metals, unremoved protecting groups or any combination thereof.

**Table 1:** Example peak assignment output (sequence and mass spectrum shown in Fig. 3). For each peak specified by the user, a set of all possible combinations of residue deletions and/or adducts agreeing with that *m*/*z* value are provided, in addition to a calculated mass for each suggestion

Peak (*m*/*z*)	Deletion(s)	Adduct(s)	Calculated mass
1063.7	R, S	Na^+^	1063.52
1197.8	S	–	1197.64
1315.9	R, S	Na^+^, Pbf	1316.52
1450.0	S	Pbf	1450.64
1537.0	–	Pbf	1537.67

A number of calculated parameters specific to peptides are also available. Peptide Calculator will provide estimated values for sequence isoelectric point and molar extinction coefficient (at 280 nm), as well as a pie chart summarizing proportions of acidic, basic and uncharged residues in the sequence (Fig. 2). For sequences that are 10 residues or longer in length, a *β*-strand contiguity profile is calculated (Fig. 2). This provides an ab initio prediction of the location of *β*-strand forming regions within the sequence, and hence may offer an indication of aggregation-prone sequences, or those which are likely to present difficulties during synthesis.

## Methods

Peptide Calculator and Peptoid Calculator make use of a database each containing either amino acids or peptoid building blocks defined by residue codes (single- or multiple-letter) and accompanying molecular formulae. Molecular weights are calculated by reference to a table of atomic masses (most abundant isotope). Methods used to generate other calculated parameters are described below. All Pep-Calc.com functionality is scripted using an extensible framework written in the Python programming language, and the site is accessed using an HTML web interface. Residue formulae can be added to either database upon request.

### Isoelectric point and molar extinction coefficient calculation

Theoretical peptide isoelectric points are calculated using the bisection method described by Kozlowski [17–19]. The net charge of the peptide can be found using the Henderson–Hasselbalch equation, taking into account contributions from negatively and positively charged groups (first and second terms in Eq. (1) respectively, where *K*_*a*_ is the acid dissociation constant of the amino acid).

1 $$charge = \sum \frac{-1}{1 + 10^{pK_a - pH}} + \sum \frac{1}{1 + 10^{pH - pK_a}}$$

As the isoelectric point (pI) represents the pH at which the net charge of the peptide equals zero, finding the root of this equation (in this case numerically, using the bisection method) gives the pI (or pH at zero charge).

Peptide Calculator takes into account side chain charge contributions from Arg, Asp, Cys, Glu, His, Lys and Tyr residues, in addition to the N-terminal amine and C-terminal carboxyl groups (only if the terminus types are set to ‘Unmodified’ and ‘Acid’ respectively). Other residue side chains are not taken into account for pI estimation, and are designated ‘Other’ in the charge summary pie chart.

Molar extinction coefficients are estimated using Eq. (2), described by Pace et al. [20]. The formula takes into account numbers of Trp and Tyr residues in the peptide ($n_{Trp}$ and $n_{Tyr}$ respectively), in addition to the number of *cystine* residues ($n_{cystine}$) formed via disulfide bond formation between pairs of cysteine side chains (reduced cysteine residues do not contribute significantly to the absorbance above 275 nm [20]).

2 $$\varepsilon _{280} \; \hbox {M}^{-1}\hbox {cm}^{-1} = 5500n_{Trp} + 1490n_{Tyr} + 125n_{cystine}$$

Peptide Calculator outputs two values for $\varepsilon _{280}$, calculating the theoretical molar extinction coefficient based on either formation of the maximum number of disulfide bonds possible ($n_{cystine}$ equal to the number of cysteine residue *pairs*), or complete reduction resulting in the absence of disulfides ($n_{cystine} = 0$).

### Automatic mass spectral peak assignment

**Fig. 4:**
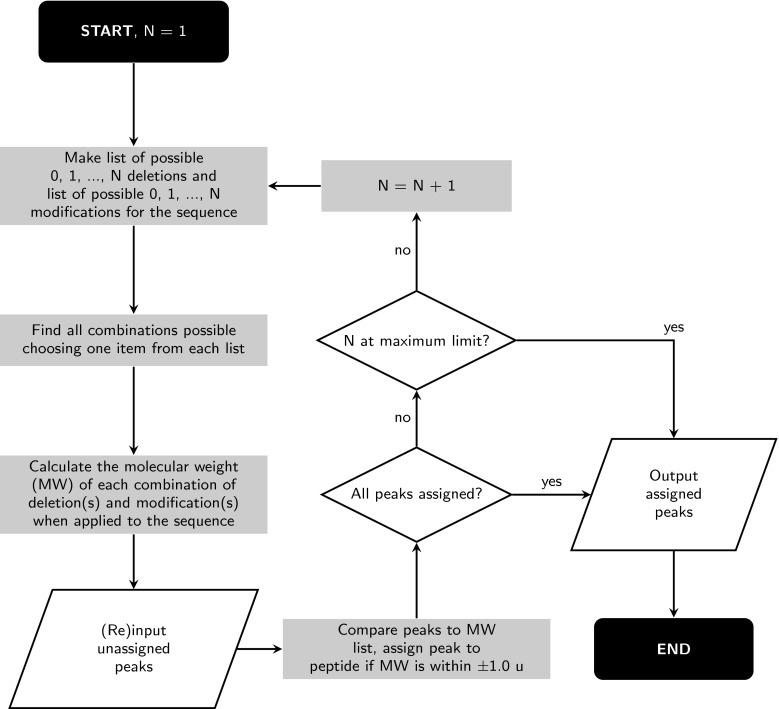
Flowchart summarizing the mass spectral peak assignment algorithm used by Peptide Calculator and Peptoid Calculator. Residues missing from the expected full sequence are termed ‘deletions’ and any other atom or group that causes a change in the molecular weight of the sequence (including metal adducts and unremoved protecting groups) is termed a ‘modification’. Which deletions and modifications are allowed depends on the residues present in the sequence (unremoved Pbf protecting groups, for example, are only permitted for Arg residues). Only sequences bearing a single deletion and/or a single modification are considered on the first iteration (N = 1), increasing to two of each on the second etc. The maximum allowed value for N depends on the length of the input sequence, and is set at 5 iterations for sequences up to and including 30 residues in length, 4 up to 60 residues and 3 up to the maximum 150 residues

User-entered *m*/*z* values are assigned through the process summarized in the flowchart given in Fig. 4. Pep-Calc first compiles lists of possible single-amino-acid deletions and single modifications (metal adducts and unremoved protecting groups), including null entries for no deletion or no modification. A complete set of combinations of these lists is then generated, and the molecular weight of the peptide/peptoid sequence incorporating each combination of single deletion and/or single modification calculated. Each input peak is then compared against the list of molecular weights, and a peak is assigned to a particular peptide if it falls within ±1.0 u of the calculated molecular weight of the peptide.

In the event that all the input peaks are not assigned on the first pass, Pep-Calc calculates the molecular weights for all peptide/deletion/modification combinations incorporating single *or double* deletions and single *or double* modifications, and checks remaining peaks against these (omitting already assigned peaks). This process is repeated until all peaks are assigned, or until up to the maximum allowed number of deletions/modifications have been checked. To prevent excessive computation times the maximum number of deletions/modification depends on the sequence length, and is set at 5 iterations for sequences up to 30 residues in length, 4 for 60-mers and 3 up to the maximum 150 residue sequence input.

### Calculation of sequence *β*-strand propensity

*β*-Strand contiguity profiles for peptides greater than 9 residues in length are calculated using an implementation of the simple algorithm for sliding averages (SALSA) described by Zibaee et al. [21]. A window of size 4 residues is scanned across the input sequence and each fragment within the window scored using Eq. (3), where $P_{\alpha },\, P_{\beta }$ and $P_{t}$ are the Chou–Fasman secondary structure probability parameters (for *α*-helix, *β*-strand and reverse turn preference, respectively) [22]. This process is repeated for all window sizes up to 20 residues or the sequence length (whichever is reached first), and all fragments with scores lower than 1.2 are discarded.

3 $$fragment \; score = \frac{\sum P_\beta }{\frac{1}{2} \left( \sum P_{\alpha } + \sum P_{t}\right) }$$

*β*-Strand propensity values are then calculated for each residue in the sequence by summing the scores of all remaining windows which contain the residue. These final values are then plotted to produce a *β*-strand contiguity profile for the peptide. Chou–Fasman parameters are only available for the 20 canonical amino acids and hence only these are taken into account when calculating *β*-strand propensity values.

It should be noted that *β*-strand propensity alone may not be indicative of aggregation likelihood or sequence difficulty. In addition, ab initio secondary structure prediction methods based on probability parameters alone can in some cases give false predictions or fail to predict regions of a given secondary structure. SALSA was chosen with speed in mind, and for this reason the calculated profile is intended to serve only as a guide.

## Conclusions

Peptide Calculator and Peptoid Calculator form a set of full featured, freely available web utilities for peptide and peptoid molecular property calculation and mass spectral peak assignment. Modern peptide research demands tools that can handle residue types beyond the canonical amino acids (such as phosphorylated peptide building blocks [23–25]), and with unique spectral assignment capabilities and an expanding amino acid database Peptide Calculator offers a service beyond that of current freely available web utilities. Furthermore, similar services for peptoid research are non-existent, and Peptoid Calculator represents a first-of-a-kind resource for researchers in the field of peptoid science. The tools described have found broad application in our lab, and are used frequently in peptide and peptoid research activities [26–28]. It is anticipated that Pep-Calc.com (http://www.pep-calc.com) will act as a valuable asset to those synthesizing and/or using peptides or peptoids as part of their research in the biophysical and life sciences fields.
